# Reducing the duration of untreated psychosis and its impact in the U.S.: the STEP-ED study

**DOI:** 10.1186/s12888-014-0335-3

**Published:** 2014-12-04

**Authors:** Vinod H Srihari, Cenk Tek, Jessica Pollard, Suzannah Zimmet, Jane Keat, John D Cahill, Suat Kucukgoncu, Barbara C Walsh, Fangyong Li, Ralitza Gueorguieva, Nina Levine, Raquelle I Mesholam-Gately, Michelle Friedman-Yakoobian, Larry J Seidman, Matcheri S Keshavan, Thomas H McGlashan, Scott W Woods

**Affiliations:** Department of Psychiatry, Yale University, 34 Park Street, New Haven, CT 06519 USA; Connecticut Mental Health Center, 34 Park Street, New Haven, CT 06519 USA; Commonwealth Research Center, Massachusetts Mental Health Center Division of Public Psychiatry, Beth Israel Deaconess Medical Center, Department of Psychiatry, Harvard Medical School, 75 Fenwood Road, 5th Floor, Boston, MA 02115 USA; Yale Center for Analytical Sciences (YCAS), 300 George Street, New Haven, CT 06511 USA

**Keywords:** Early intervention, First-episode services, Duration of untreated psychosis, Schizophrenia, First-episode psychosis, Public health campaigns, Health communication campaigns

## Abstract

**Background:**

Early intervention services for psychotic disorders optimally interlock strategies to deliver: (i) Early Detection (ED) to shorten the time between onset of psychotic symptoms and effective treatment (i.e. Duration of Untreated Psychosis, DUP); and (ii) comprehensive intervention during the subsequent 2 to 5 years. In the latter category, are teams (‘First-episode Services’ or FES) that integrate several empirically supported treatments and adapt their delivery to younger patients and caregivers. There is an urgent need to hasten access to established FES in the U.S. Despite improved outcomes for those in treatment, these FES routinely engage patients a year or more after psychosis onset. The Scandinavian TIPS study was able to effectively reduce DUP in a defined geographic catchment. The guiding questions for this study are: can a U.S. adaptation of the TIPS approach to ED substantially reduce DUP and improve outcomes beyond existing FES?

**Methods/Design:**

The primary aim is to determine whether ED can reduce DUP in the US, as compared to usual detection. ED will be implemented by one FES (STEP) based in southern Connecticut, and usual detection efforts will continue at a comparable FES (PREP^R^) serving the greater Boston metropolitan area. The secondary aim is to determine whether DUP reduction can improve presentation, engagement and early outcomes in FES care. A quasi-experimental design will compare the impact of ED on DUP at STEP compared to PREP^R^ over 3 successive campaign years. The campaign will deploy 3 components that seek to transform pathways to care in 8 towns surrounding STEP. Social marketing approaches will inform a public education campaign to enable rapid and effective help-seeking behavior. Professional outreach and detailing to a wide variety of care providers, including those in the healthcare, educational and judicial sectors, will facilitate rapid redirection of appropriate patients to STEP. Finally, performance improvement measures within STEP will hasten engagement upon referral.

**Discussion:**

STEP-ED will test an ED campaign adapted to heterogeneous U.S. pathways to care while also improving our understanding of these pathways and their impact on early outcomes.

**Trial registration:**

ClinicalTrials.gov: NCT02069925. Registered 20 February 2014.

## Background

### The promise of early detection

Psychotic disorders are a source of severe distress, disability and economic cost under usual systems of care. The prototypic schizophrenia spectrum disorders affect between 0.55% and 1% of people during their lives [1]; and typically manifest as a ‘first-episode’ of psychotic symptoms in adolescence or early adulthood. This is an especially formative period for social and vocational trajectories and psychotic disorders are among the top 20 causes worldwide of years lived with disability (YLD), exceeding epilepsy, ischemic heart disease and Alzheimer’s disease in their impact amongst the chronically ill [2]. With routine care, less than one-fifth of patients achieve full recovery after a first episode of psychosis [3] and less than one-third achieve minimal age-appropriate employment or education [4]. Chronic psychotic disorders, while relatively less prevalent, lead annual U.S. expenditures on mental illnesses, with $22.7 billion in direct healthcare costs, and $32.4 billion in indirect costs stemming from unemployment, reduced workplace productivity, premature mortality from suicide, and family caregiving [5].

Early intervention can significantly ameliorate the poor outcomes of usual care. The first 2–5 years after psychosis onset presage much of the eventual clinical and psychosocial deterioration in schizophrenia spectrum disorders [6] including one-third of completed suicides [7], relapse and re-hospitalizations [8], violence [9] and neurocognitive dysfunction [10]. This period also offers the opportunity to modify several prognostic factors including substance misuse, social isolation, negative symptoms and cognitive dysfunction [11]. These factors can derail an already vulnerable period of emerging adulthood [12]. Relapses in the first year or two after the first episode, and accumulated duration of positive symptoms of psychosis in the first few years of illness are associated with worse neurocognition 5–10 years later [13]. The hypothesis of a ‘critical period’, wherein secondary prevention efforts 2–5 years after psychosis onset might disproportionately leverage long term functional gains, [14] has stimulated a rich variety of approaches to early intervention.

Early Intervention (EI) for psychotic disorders can be conceptualized as including interlocking strategies to deliver: (i) Early Detection (ED) to shorten the time between onset of psychotic symptoms and effective treatment (i.e. Duration of Untreated Psychosis, DUP); and (ii) comprehensive intervention during the 2 to 5 years after identification of a psychotic disorder [15]. Of particular promise, in the latter category, are teams (henceforth ‘First-episode Services’ or FES) that integrate several empirically supported treatments and adapt their delivery to younger patients and their caregivers. FES have demonstrated improvements in symptoms, functioning and costs in a range of healthcare systems [16,17]. The two collaborating FES in this project (clinic for Specialized Treatment Early in Psychosis, or STEP in New Haven and Prevention and Recovery in Early Psychosis, or PREP^R^ in Boston) have successfully applied comparable FES in similar settings to deliver feasible, sustainable and effective care [18,19]. STEP recently completed the first U.S. randomized controlled trial to determine the feasibility and effectiveness of this public-sector based approach to FES [18]. This trial has also collected detailed information on sources of direct and indirect cost [20–22] that support international data on the health economic benefits of FES.

There is an urgent need to hasten access to FES in the U.S. Despite improving outcomes for those in treatment, STEP and PREP^R^ continue to admit patients long after psychosis onset. For example, over half (51%) of all entrants to STEP over the past 6 years had endured a DUP longer than 3 months with evidence of significant morbidity on arrival to the service: about 10% of entrants had already attempted suicide, 45% had a co-morbid substance use disorder and 60% had dropped out of school or employment while 90% entered care after a behavioral crisis requiring hospitalization [23]. The same proportion (50%) had endured a similarly prolonged DUP over the past 5 years at PREP^R^.

Early Detection has the potential to substantially reduce DUP and extend the impact of existing FES. The largest experimental study of ED has been the TIPS study. TIPS delivered a combination of public and targeted education campaigns coupled with rapid availability of FES in 2 healthcare sectors in Norway, whereas two control sectors (in Norway and Denmark) delivered comparable FES without the benefit of these efforts to reduce DUP. This strategy more than halved DUP (from a median of 15 to 4.5 weeks) [24] at the ED sites. This DUP reduction was tied to markedly reduced distress and disability at clinical presentation, including reduced positive and negative symptoms of psychosis [24] and suicidal behavior [25]; persistently improved negative symptoms at 5 years [26] and resulted in twice the rate of recovery (31 vs. 15%) at 10 years [27]. Notably, when the information campaign was interrupted due to lack of continued funding, this resulted in a decay of the DUP toward the control levels followed by attendant worsening in outcomes [28]. This validated a causal role for DUP in affecting outcomes rather than functioning solely as a marker of poor prognosis subgroups [29]. Although one other study in Singapore used a similarly broad, multifocal campaign to reduce DUP and improve pathways to care, [30] other less comprehensive attempts have failed to reduce DUP [31], demonstrating the challenge of replicating the promising findings from TIPS.

Several factors argue for careful study rather than straightforward application of the TIPS approach to U.S. settings. Pathways to specialty care in the U.S. are diverse, potentially weakening some but also magnifying the impact of other efforts toward early detection. Also, the anticipated but unclear changes in U.S. pathways over time (e.g. with ongoing reforms in access to health insurance [22]) make it vital to test any ED strategy against a control setting to disentangle the effects of an ED campaign from secular changes in the U.S. healthcare environment. Finally, FES care is a rarity in most U.S. settings and can reduce DUP in a region simply by its presence. Controlling for this effect across 2 established FES can a) help disentangle the impact on DUP of a distinct ED campaign and, b) if the ED campaign is successful, clarify the added value of optimizing timing (ED) over already existing efforts to improve the quality of early treatment (i.e. FES).

### Contextualizing early detection within pathways to care

**Figure 1:**
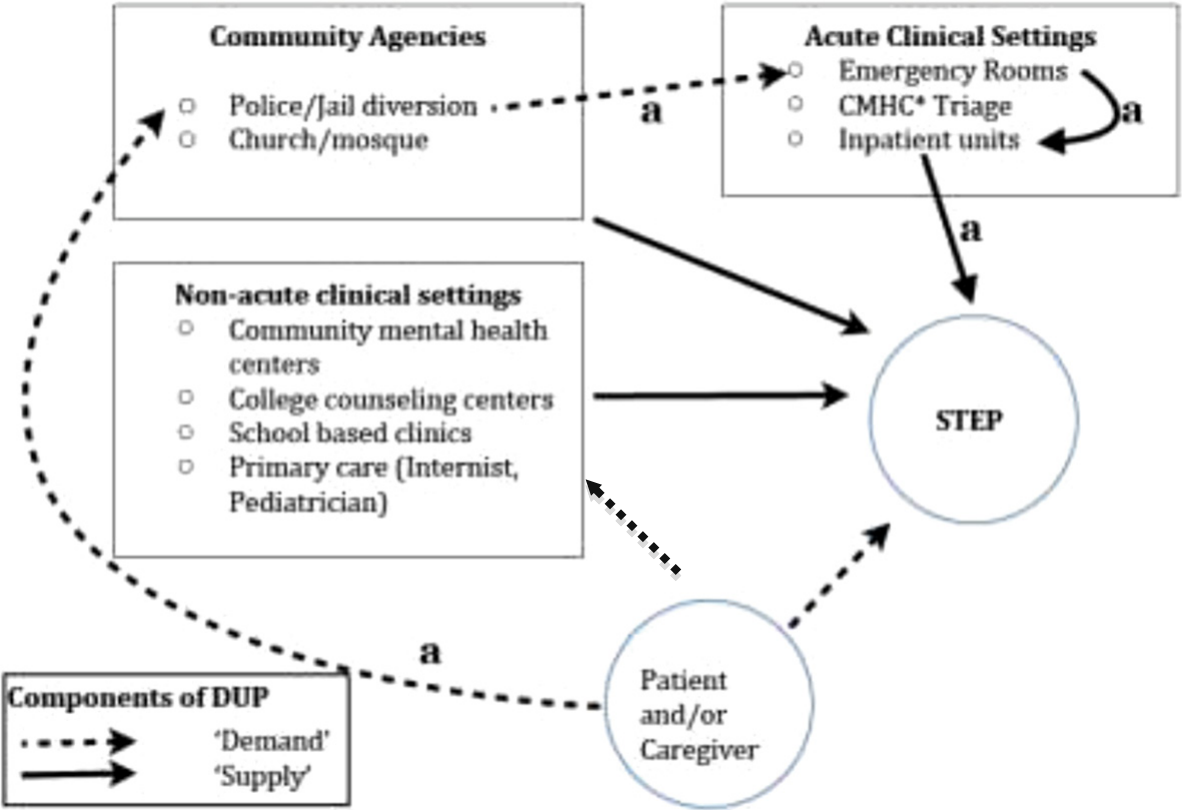
**Heuristic model for DUP in STEP’s catchment.**

Our attempt to reduce DUP in the complex U.S. healthcare environment uses a model (Figure 1) that (a) incorporates local knowledge and uncertainty of the size and determinants of various sources of delay and (b) the pathways to care that reveal potentially malleable nodes on which to prioritize efforts. We prefer to categorize the determinants of DUP as including a ‘demand’ side (e.g. delays in effective help-seeking resulting from factors including delays in illness identification and delayed attempts to access healthcare by patients and caregivers) [32] and a ‘supply’ side (delays by professionals in initiating treatment that include delays in diagnosing and initiating or referring to appropriate treatment). Given the variability of impact of sources of delay across various U.S. settings, our approach is agnostic on whether to prioritize demand or supply side interventions. We begin with the premise that deploying interventions targeting these two domains of DUP with already demonstrated success (in the TIPS project) provides an empirically tractable basis for the main goal of DUP reduction while also allowing for vital information to be collected on determinants of DUP within the context of local pathways to care.

Pathways to care can be defined as “the sequence of contacts with individuals and organizations prompted by the distressed person’s efforts, and those of his or her significant others, to seek help as well as the help that is supplied in response to these efforts [33]”. This highlights the interleaved behaviors of help-seeking by patients and families on the one hand, and the response of professionals and agencies on the other in generating a particular patient’s pathway into treatment. There is considerable information on a diverse set of barriers relevant to U.S. pathways, but the lack of consensus on measures and the multiplicity of factors limits straightforward application of available measures to a particular geographic area. Fortunately, STEP has collected relevant information over 6 years of recruiting from the local catchment that has included more than 600 formal inquiries and multiple informal consultations to families, clinics and area non-healthcare settings (e.g. churches, schools, mosques). This has allowed us to populate a heuristic model. While not exhaustive, it integrates salient elements of our local pathway to care.

We integrate these ideas in a modified version of the influential Goldberg-Huxley model [34] (Figure 1) to understand the interaction of both patient/caregiver factors and system factors relevant to DUP. The choices for sampling, ED implementation, measurement and analysis, described in the succeeding sections, derive from this model. While not exhaustive, the boxes correspond to the most common ‘levels’ or recognized sources of care in STEP’s target catchment (8 towns). The model conceives of these levels as not just sources of referral or delay but rather as ‘filters,’ hastening access to STEP for some kinds of patients while potentially delaying it for others. This phenomenon of filtering, whereby different levels of care may influence access in non-random ways, is exemplified by STEP’s experience of rapid (short-DUP) referrals of agitated, predominantly male patients from acute settings on the one hand, while also explaining a paradoxical finding reported in other settings: prior connection to outpatient care may actually delay entry to specialty FES [35]. Particular kinds of patients may proceed through particular ‘levels’ of care with unexpected speed or delay and this suggests the need to target, measure and analyze sources of DUP within the context of local pathways.

Pathways to care can be usefully conceptualized, from the perspective of the patient or caregiver, across 4 principal events: the onset of psychotic symptoms, a recognition of a need for care with related help-seeking behavior, an encounter with a professional agency and finally, entry into specialty first-episode care. Within our model, the sources of delay are best illustrated by the example depicted by the pathway taken by one patient (‘**a**’) to the STEP clinic and depicted by the arrows in Figure 1:

- VS suffered the onset of psychotic symptoms 4 months before his mother decided to call the police after he pushed her to the floor and threatened her with a kitchen knife. The police officer found him to be disheveled, loudly alleging that “[the neighbors are] messing with my business” and that his mother “does not believe me.” The officer had to threaten VS with arrest to get him to agree to an evaluation in the local emergency room. He arrived in the hospital upset and refusing to cooperate with the psychiatric evaluation, at one point requiring soft leather restraints and involuntary intramuscular medication after he threw his food tray at a staff member and attempted to run away. After a brief hospitalization of four days during which he agreed to take antipsychotic medications, and follow-up with aftercare, he was discharged with a referral to the STEP clinic. VS did not show for his first appointment at STEP and when called at home indicated he did not wish to seek further care. After 2 more involuntary hospitalizations under similar circumstances and his mother’s insistence that he return to STEP in order to be able to continue to live at home, he arrived for his first outpatient appointment, 8 months after the onset of psychotic symptoms. Another month elapsed before VS agreed to resume antipsychotic medication and engage with his primary clinician in developing a treatment plan that focused on his goals of returning to community college and finding part-time employment. He remains unwilling, six months later, to explicitly acknowledge the role of a psychotic illness in his difficulties, but is adherent to medications and working with a supported employment specialist to develop his resume for competitive employment.

The story illustrates several salient contributors to DUP. The delayed recognition of the presence of an illness by the patient and his mother reflects a combination of factors including lack of awareness of the signs of mental illness and of available resources, denial, and fear of stigma - all of which delay effective help-seeking behavior that could have preempted the behavioral crisis and police intervention. The aversive experience of entering and receiving care under duress likely reduced the patient’s willingness to follow through on referrals to an FES. Many other combinations of the various levels are involved in the variety of pathways that contribute to the ‘demand’ and ‘supply’ components depicted in Figure 1. It is important to note that the demand and supply contributors to DUP often do not resolve in a linear fashion, but can result from a dynamic interplay of events. For example, a patient may enter an FES at the behest of a parent, with effective interruption of the DUP long before they can identify the presence of symptoms or the need for professional care. This perspective informs the pluralistic strategy of the campaign detailed below, which envisions diverse ways to interrupt DUP that seek to facilitate, but do not require, salutary changes in attitudes, beliefs or stigma.

The STEP-ED campaign will target both the lack of effective ‘demand’ for specialty care (dotted line) and effective ‘supply’ of such care (solid lines) in 3 principal ways: (1) a public Education Campaign (PEC), (2) outreach to and academic detailing of professionals (PO) to modify the filtering enacted at the various levels depicted in the model and (3) implementing Rapid Access to STEP (RAS) that will include a variety of performance improvement tactics to reduce the time between referral and admission to STEP.

### Aims and Hypotheses

This study seeks to test of the effectiveness of an early detection campaign in a policy-relevant U.S. setting, where relatively fragmented pathways to care magnify both the challenges and the potential public health impact. The presence of 2 similar, effective, geographically separated but collaborative FES programs (STEP and PREP^R^) presented an excellent opportunity to conduct such a test and thereby advance secondary prevention for psychotic illnesses in the U.S.

The guiding questions for this study are: can a U.S. adaptation of the TIPS approach to early detection substantially reduce DUP and improve outcomes beyond existing FES?

#### The primary aim is

To determine whether early detection intervention can reduce DUP in the US, as compared to usual detection. Early detection (ED) will be implemented in one US community (New Haven, CT), and usual detection efforts will continue in another (Boston, MA). DUP will be measured at admission to the corresponding services (STEP & PREP^R^) in each community, over one year before and throughout ED implementation. We hypothesize that DUP will be reduced significantly in the early detection site compared to the usual detection site.

#### The secondary aim is

To determine whether DUP reduction can augment the outcomes of established FES. We will measure symptoms, functioning and engagement with treatment at entry and over 1 year at each site. We hypothesize that the shorter DUP engendered by the ED campaign at one FES (STEP) will result in reduced distress and illness severity at entry and better 1 year outcomes and engagement at STEP vs. PREP^R^.

## Methods/Design

### Design rationale

Any effort to test the impact of an early detection strategy to reduce DUP and determine the subsequent impact on early outcomes, must confront specific biases [36] that are each addressed in the Procedures detailed below. First however, the overall rationale for the quasi-experimental design involving 2 sites is outlined:

### Primary aim

Any test of a DUP reduction strategy in a community has to consider 2 main sources of confounding. First, unexpected changes in either direction for DUP may occur over the life of this study (e.g. variation in healthcare access due to macroeconomic changes) that will imperil inferences from the sole use of a historical control to determine the impact of ED on DUP. The parallel control in this proposal (PREP^R^) ameliorates this concern by being carried out during the same period and allowing for careful measurement (see below) of these influences. In addition, the historical control design in New Haven (pre- and post-ED) also allows us to consider the effects of ED without the impact of unknown sources of heterogeneity in DUP across sites. The second source of confounding is preferential sampling from the larger target sample of new onset cases. For example, a correlation between ED and shorter DUP might merely reflect strategic success at diverting short DUP cases to STEP, while longer DUP cases remain untreated, or treated elsewhere, in the community. This is addressed by (a) our intensive media and targeted education campaign to facilitate representative recruitment from across the catchment and (b) the availability of a historical control at the STEP site, wherein DUP distributions available for years before ED implementation will allow pre-post comparisons to assess for differential skewing. Finally, (c) DUP will also be measured at a major community mental health center (the Hill Health Center, HHC, Connecticut) in the catchment area of the ED intervention for one year before and during the ED implementation. Specifically we will measure DUP for those who are not successfully referred to STEP. Three possibilities will be considered for this group: if DUP at HHC remains the same or falls, this would reassure us that ED had no effect or a salutary effect on help-seeking, even if not to STEP; while if the DUP rises for this group, it would raise concerns for biased sampling as described above. Notably, such skewed sampling did not occur in the TIPS project [37] and given the similar catchment based design, and the reality that few competing resources for early psychosis care are available in the STEP catchment, such skewing is unlikely to occur in STEP-ED.

### Secondary aim

Disentangling the additional benefit of DUP reduction on the early outcomes of FES care would ideally be determined by randomized allocation of ED vs. no-ED within a single sample that would all subsequently receive the same FES care. This is fraught with logistical challenges. Any effective public education campaign would be hard to isolate to only some members of a catchment. The next best alternative is the quasi-experimental controlled design proposed here, with careful assessment (see Measurement) and statistical adjustment (see Analysis) for inevitable sample differences with equivalent FES care (STEP or PREP^R^) at two geographically separated sites. A residual bias is that a correlation of DUP with better 1 year outcomes at the ED site (i.e. STEP) does not rule out the possibility that better (or worse) prognosis cases were easier to detect and enter into FES early [38] increasing the chances of a Type 1 (or 2) error, respectively. For example, patients with insidious-onset, deficit symptom ridden illnesses (with generally poorer prognoses) may be quietly psychotic for years before coming to therapeutic attention whereas good prognosis patients with sudden and visible positive symptom related alterations in behavior may alert observers and come to the attention of health providers and preferentially enter STEP. Conversely, the acute, rapidly resolving psychotic disorders (with better prognoses) may be less likely to be discovered by our early detection campaign than those who are continuously symptomatic in the catchment [39]. Having a parallel control site with careful measurement of diagnostic profiles and other prognostic factors at each site (see Measurement) will allow us to limit the impact of this bias. Also this will allow analysis for the possibility that DUP is, for different subgroups, both a malleable mediator of early outcome and a marker for those subgroups who are less responsive to FES [40].

Additional biases related to patient refusal (e.g. longer DUP patients tending to decline participation in the TIPS study [41]) and unanticipated exclusions will also be measured and adjusted for in the analysis.

### Sampling

The early detection strategy (ED) will target all residents of a designated geographic catchment contiguous with the Connecticut Mental Health Center (CMHC) in which STEP is located. This includes the 8 towns of Bethany, Orange, Woodbridge, Hamden, New Haven, East Haven, West Haven and North Haven, with a total population of 323,285 (Census, 2010) and for which we estimate an annual incidence 40–70 cases of schizophrenia-spectrum disorders. The catchment was chosen based on feasibility of travel for care at STEP. The intensive approach to ascertainment of new onset cases from across the catchment will include public messaging and targeted outreach to all major treatment centers. Also measurement of DUP will be undertaken at one other major community mental health center that draws from this catchment zone. At this community ‘sentinel’ site, in addition to the usual outreach, clinical records will be reviewed on a regular basis to determine the prognostic profile and DUP of patients who were not successfully referred to STEP for initiation of treatment. The control site, PREP^R^ is based in the Jamaica Plains area of Boston and while it draws from a much larger metropolitan population of more than 4 million (Census 2010), the demographic and prognostic profile of usual recruits over the past 5 years have been broadly comparable to that of STEP (see section *Choice of Control site* below).

The focus of early intervention after the onset of a psychosis is to improve the outcomes of individuals with schizophrenia-spectrum disorders. The reality, embraced by all exemplar early intervention programs, is that an accurate diagnosis is often difficult to make at the time of symptom onset [42]. Thus a ‘first-episode’ psychosis sample will necessarily include patients who will, on longitudinal follow-up, turn out to have less severe illnesses such as major depression with psychotic features, brief psychotic disorder or bipolar disorder (with psychotic features). The study population is thus expected to be diagnostically heterogeneous in the service of identifying, with as much sensitivity as possible, those who are likely to develop chronic psychotic illnesses.

We will thus use criteria that are simple to communicate and apply, to minimize delays in determining eligibility:

- Inclusion Criteria: 16–35 years old, must live within catchment of interest (For PREP^R^: greater Boston metropolitan area; for STEP: 8 town catchment) and must have had their first-episode of psychosis within the past 3 years.
- Exclusion Criteria: Established diagnosis of Affective psychosis (i.e. non-ambiguous Bipolar d/o or MDD with psychotic features) or Psychosis secondary to substance use or a medical illness, unable to communicate in English, eligible for DDS (Department of Developmental Services), legally mandated to enter treatment, unable to reliably determine DUP, unstable serious medical illness. We will exclude from this study, those patients who converted to psychosis while being followed and cared for in prodromal clinics (i.e. DUP of 0), which exist at both sites. We will also exclude those who have previously received care at another FES.

Reasons for exclusion and patient refusal will be recorded for all referrals. DUP will be estimated for refusers who are otherwise eligible, to gauge the impact of sampling bias on the relationship between DUP reduction and early outcomes.

Written informed consent for participation in the study will be obtained from all adult participants. For those participants under 18 years of age, written consent will be obtained from a parent or legal guardian in addition to written assent from the participant. All procedures are in compliance with the Helsinki Declaration and have received ethics approval from the Yale University Human Investigation Committee (Protocol Number: 1310012846).

### Intervention: the early detection campaign

#### Theory of the campaign

The STEP-ED campaign seeks to reduce all malleable sources of delay that contribute to the duration of untreated psychosis (DUP). As noted above, we conceive of two broad sources of delay. The first refers to the ‘Demand’ (identification of illness and initiation of help-seeking) for care and the second covers events that refer to the ‘Supply’ of the needed care (correct identification of diagnosis and referral to first-episode services).

To intervene on the Demand side, the campaign will transmit messages to hasten identification (by the patient, caregiver or friend) of a need for professional assistance and enable effective help-seeking behavior. There is a considerable body of literature with a number of models that emphasize different approaches to inducing such changes in health behavior. However, empirical testing of specific interventions based on these models has been inconclusive [43]. For example, there was no difference between messaging based on the Theory of Reasoned Action and general informational messaging in encouraging young men to perform testicular self-examinations [44]. A test of the influential Transtheoretical Model of Change, found that interventions carefully matched to an individual’s stage of change were actually less effective than mismatched interventions for smoking cessation [45]. Furthermore, these theories can be criticized for de-contextualizing the process of acting to take care of one’s health, treating individuals as ‘islands’ of cognitive and deliberative processes. This reduced their face validity for disorders that are marked by poor insight.

In contrast, the STEP-ED campaign will employ an ecological approach. Demand-side events (such as identifying that there is a problem) including internal cognitive processes in a patient or family member, are expected to routinely interact with Supply-side events, such as the responses of service providers and other helpers in the community. This ecological approach targets networks [46] of potential first responders who determine patients’ ‘pathways to care’ which do not solely or even substantially rely on putative drivers of individual health behavior that have been posited in extant models [43]. A modified Goldberg-Huxley model of pathways to care (introduced in Contextualizing early detection within pathways to care) highlights the interaction of patients/families with various care providers and introduces the concepts of ‘levels’ of care and ‘filters’ of care that is expanded in our approach beyond traditional clinical settings to include community agencies or non-clinical actors who are influential in determining DUP. While ‘levels’ can be conceived of as the different agencies that deliver different grades of expertise at different stages and needs of the illness, ‘filters’ refers to the active role these agencies might play in differentially modifying DUP for different sub-groups or patients or different presentations of illness/need.

While we assume that all of the actors in our local pathways to care (see Figure 1) may, for different patients, play a causal role in DUP and are thus part of our model, we do not presume to attribute weights to their relative importance. Research in other systems of care that have employed selective campaigns (e.g. focused only on the Supply side by educating GPs) suggests that a broader ‘kitchen-sink’ [47] approach that is agnostic about, and treats their relative weights as an empirical question (especially within the more fragmented US healthcare ecology) is more likely to achieve DUP reduction. The STEP-ED campaign, will therefore be pluralistic and implement three interlinked areas relevant to DUP.

#### Components of the campaign

The first year of the study period will be used to develop the campaign. The broad outline of the 3 components is depicted in Table 1. While the theory and structure or overall strategy of the campaign are described above; the specific tactics, including messaging, choice and use of channels of dissemination and real-time adaptations will employ novel social marketing approaches. Specifically, this will include development of a campaign brand image and logo, the use of analytic tools (e.g. Google analytics) to assess online traffic related to STEP-ED’s message disaggregated by target sub-populations (e.g. age, gender, town of residence) with the ability to course correct the targeting of messages. While traditional ‘mass media’ channels will also be deployed, the advantage of social media will be exploited to achieve comparable and more youth appropriate mass circulation while retaining the ability to personalize the message and have it amplified in a cost-effective manner (e.g. sharing via peers on Facebook).

**Table 1:** **Components of STEP-ED campaign**

**A. Public education campaign (PEC)** with messages targeting: (i) patients; (ii) parents/caregivers and (iii) friends to empower them to facilitate entry into care. Will include the following channels of communication:	(1) Social media (e.g. Facebook, Twitter, YouTube)
	(2) Mass media: including radio, (limited) TV, newspapers, mailings
	(3) Interactive campaign website
	(4) Posters on buses
	(5) Promotional items
**B. Professional outreach & detailing (POD)** with distinct strategies targeting:	(1) Educational settings (College counseling, School Based Health Clinics)
	(2) Mental health services (Public and private, agency and solo practice)
	(3) Primary care settings (Adult and Pediatric)
	(4) Religious organizations
	(5) Consumer organizations
	(6) Judicial system
	(7) Policy and Legislative actors
	(8) Social Welfare Agencies
**C. Rapid Access to STEP (RAS)** including:	A variety of performance improvement measures focused on reducing time between referral and entry into treatment at the STEP clinic.

The campaign development process and final details of all 3 components will be available in a forthcoming paper, but the overall structure is summarized below:

##### Public Education Campaign (PEC)

The target population of the PEC is those individuals with early psychosis, and their caregivers or friends, who are yet to make contact with one of the levels of care. Messaging will be developed to enable effective help-seeking by providing specific, actionable, and persuasive messages on (1) how to identify the signs and symptoms of psychosis; (2) how to access professional evaluation and effective care at STEP; and (3) the effectiveness of early treatment. While some of the early message development will include feedback from focus groups, real-time feedback and refinement will be a key feature, with the use of online tools to track visits to social media and web sites. The channels for the media campaign will include television, radio, bus ads, a website, social media (e.g. Facebook, Twitter, & YouTube), promotional items, and print materials for professionals to share with at-risk youth and parents.

##### Professional Outreach and Detailing (POD)

Professionals in the 8 levels posited to influence pathways to care in our area (see Figure 1) will be targeted via interpersonal outreach (face-to-face meetings, workshops, and phone calls) and resource materials (print and web-based) followed by a focus on careful detailing of key referral sources identified during initial outreach. In keeping with evidence from the limited literature on what appears to influence provider behavior [48,49] we will use detailing as a way to both understand barriers and enable actions that will successfully and quickly divert patients to STEP for care. All materials will be designed to transmit a uniform message consistent with the PEC. Multiple stakeholders within each setting will be identified and an appropriate plan for repeated contact or detailing will be devised. For instance, the campaign targeting colleges in our catchment will include in-service sessions for Counselors followed by regular visits to their clinics to refresh the message and allow for consultation on potential referrals. The PEC will also serve as a reminder to professionals about the key points of the project and remind them of their important role in referring patients to STEP.

##### Rapid Access to STEP (RAS)

Seamless access to STEP will be an important component of reducing supply side contributors to DUP. Careful coordination will also be maintained with the Connecticut-wide 24 hr warm line (211) to route all potential early psychosis referrals to STEP. All messages to the STEP line will be cleared within 24 hours. A structured telephone screening will establish the presence of a new onset psychiatric illness within the inclusion age range and a request for help from either the adult patient or caregiver of a minor. If a potential prodromal or psychotic illness is suspected, in-person evaluation will be offered within one working day. This can begin, if necessary, in the patient’s home, school or referral setting (primary care clinic or psychiatrist office). For referrals in which the caregiver is seeking help in the face of patient reluctance or a behavioral emergency, education about available resources will be provided to the caregiver (including how to access emergency services and local parent and youth led consumer organizations) and ongoing support to problem solve and enable entry into care. In all cases, repeated attempts to contact by phone and offers to meet in the community will be made until explicit refusal from the patient, to counter the common ambivalence that can delay entry into care. When the patient consents to care, caregivers will be invited to participate in structured family education interventions (offered as a routine part of STEP and PREP^R^ care) that we expect will also facilitate the patients’ engagement into care.

#### Choice of control site

A significant strength of this design is the degree to which the 2 sites can claim to draw from equivalent samples. Relevant to this judgment are the similar cultures, recruitment and treatment philosophies at the 2 FES (STEP, New Haven and PREP^R^, Boston) that have been active over an equivalent period of time and exist within longstanding academic collaborations with State mental health agencies. Both clinics are located within diverse, urban settings and have a history of recruiting patients with comparable levels of ethnic diversity, diagnostic profiles and co-morbidity [18,19]. Furthermore, both FES are closely connected with clinics (PRIME in New Haven and CEDAR at Boston) directed by Co-Investigators on this grant that are part of the NIH-funded North American Prodrome Longitudinal Study (NAPLS). This is a consortium of clinical research centers studying ways to identify individuals who are at risk for an initial psychotic episode. Both sites have thus had a long history of community engagement around early intervention and experience with common measurement procedures.

While the prodrome clinics and FES at both sites have acquired customary referral sources and interested local stakeholders, *neither* have implemented the TIPS-style ED strategies proposed here i.e. either by intensive public education, intensive targeting of referral sources or operationalizing rapid access to their FES. Hence the early detection approach in STEP-ED will deliver a qualitative, rather than a merely quantitative change in the ED (STEP) vs. no-ED (PREP^R^) site. This will be confirmed by measures of publicity and outreach activities at both sites before and during the STEP-ED campaign.

#### Stakeholder engagement

Salient stakeholders are a robust element of this project. Specifically, message development will include input from the core audience of younger mental health consumers, caregivers and professional groups. Relevant institutional partners include the National Alliance for the Mentally Ill (NAMI), Connecticut and Massachusetts Mental Health Centers and Departments of Mental Health. Professional organizations such as the Connecticut chapters of the American Psychiatric Association, the American Academy of Child and Adolescent Psychiatry and National Association of School Psychologists have made specific commitments to assist in dissemination of the campaign message via newsletters or invitations to present at annual meetings. Additionally, an extensive network of contacts developed by the PRIME and STEP clinics over several years from invited presentations and consultations represents a vital background of strong and credible support from the multiple community health agencies and educational institutions within our catchment.

### Measurement

#### Choice of measures

##### Measuring DUP

Consensus on a valid measure of DUP has been elusive. There are difficulties in reliably determining the onset of a private mental event and also reasonable differences in opinion on what constitutes meaningful offset, or when treatment can be considered to have begun [50]. We will collect detailed onset data for a variety of psychotic phenomena and treatment events in order to allow comparison with other study cohorts. For the purposes of planning our recruitment, we chose to define onset of DUP per the threshold criteria for positive or disorganized symptoms of psychosis in the Symptom Onset Scale (SOS) [51]. The SOS was chosen for several reasons: (a) it is comprehensive, rating the severity and dating the onset of 16 general prodromal, positive, negative, and disorganization symptoms, as well as recording clinician, family, and patient global ratings of onset of illness; (b) it has good reliability; [51] and (c) both sites in New Haven and Boston have had considerable experience with this instrument over ≥5 years.

With respect to offset, we conceptualize DUP1 as ending on the date of initiation of antipsychotic medication treatment targeting psychosis. Given evidence that these symptoms can respond within a few days of antipsychotic medication treatment, [52] we chose *initiation* over more stringent criteria for *adequacy* of medication treatment proposed by others (e.g. adequate dose with 75% adherence for at least 4 weeks [53]). This will allow us to disentangle timing from quality of treatment (in which one might include the receipt of an adequate antipsychotic trial). Assessors will collect an inventory of every medication prescribed to the patient, including dates and minimal adherence to help determine the offset of DUP1. We define DUP2 as the date of admission to an FES (STEP/PREP^R^ or other comparable services in the Boston area). This approach allows us to evaluate the differential impact of the campaign on various definitions of offset [53] and subsequently, on 1 year outcomes.

##### Measuring the impact of ED on DUP

Any attempt to reduce, measure and evaluate the impact of DUP reduction within the complex U.S. healthcare environment raises challenges for disentangling the effect of the ED campaign from multiple, dynamic sources of variability in DUP. One approach would be to rely on interventions that focus on particular hypothesized mediating variables (e.g. stigma) that can then be measured and mechanistically tied to the desired outcomes. This would require assumptions about which factors would be most relevant to reduce DUP in any particular community that are unwarranted given the current level of knowledge of such factors. Indeed, while surveys or focus groups are intuitively appealing ways to elucidate these factors, the same realities that result in prolonged DUPs makes access to informative and representative samples of first-episode patients, or their families, impractical. We will instead deploy an adaptation of a previously successful approach to DUP reduction (i.e. TIPS) while also collecting detailed information about pathways to care across the two sites that will improve knowledge of factors salient to DUP. While it is possible that relative changes in these pathways at the ED vs. non-ED site may be caused by unmeasured factors that are unrelated to the campaign, the ambitious goal of halving the log-transformed DUP is intended to make it very unlikely that this could be achieved without a meaningful change in these mediating pathways.

We will use an adapted version of the Pathways to Care Interview [54] to gather systematic information (from patients, caregivers and clinical records) about the source, motivation, sequence and timing of help-seeking by patients and their families, the response offered by their first contacts for help (whether at clinical, educational, or other institutions), and the sequence and duration of subsequent contacts until entry into STEP/PREP^R^. The instrument has been modified to assist evaluation of the campaign by eliciting the sources of information that directed them toward STEP/PREP^R^. The data will allow assessment for changes in both the ‘demand’ and ‘supply’ components of DUP. Also, visual ‘route timelines’ along the modified Goldberg-Huxley model (Figure 1) will be generated to evaluate and respond to prominent sources of rapid access or delay within the catchment.

##### Measuring the impact of DUP reduction on early (1-year) outcomes

The effect of ED on early outcomes will be measured in the year following entry into treatment. While significant symptomatic improvement has already been demonstrated during this period for a majority of treated first-episode patients in U.S. settings, [8] the impact of earlier engagement with an FES is hypothesized to manifest in added gains in social and vocational functioning. A composite outcome of ‘vocational engagement’ defined as the proportion of patients who are in at least part-time school, work or better, will be derived from the Global Functioning Social and Role Scales [55]. We will also categorize patients’ vocational activity according to Bureau of Labor Statistics definitions, to allow for comparison with peers in local and national samples. We will also account for patients who are not in the labor force due to being a student or caregiver. Additional broad quality of life measures will include the Heinrichs-Carpenter Quality of Life Scale, [56] and the Short Form Health Survey (SF 36) [57].

Psychiatric diagnoses will be informed by a baseline and 1 year SCID (Structured Clinical Interview for DSM-IV) [58]. The Structured Interview for Prodromal Syndromes (SIPS), Version 5.3, given at baseline, will provide a preliminary assessment of whether the patient is at-risk for psychosis or has crossed the threshold into psychosis. It also provides additional data regarding onset dates for putative prodromal symptoms. The Global Assessment of Functioning from this measure adds a well-anchored, population-appropriate scale of overall functioning and will be repeated throughout the study. Symptoms will be measured using the Positive and Negative Symptom Scale (PANSS) [59] and the Calgary Depression Scale [60]. While violence resulting in injury to others is a rare outcome in psychotic disorders, as many as a third of patients will have committed a violent act before entering treatment and prolonged DUP has been associated with acts of serious violence [9]. The Modified Overt Aggression Scale (MOAS) [61] will be used to assesses three types of self-reported violence: physical aggression, verbal aggression and property aggression. Suicidality will be assessed with the Columbia Suicide Severity Scale (C-SSRS) [62]. Substance use will be monitored with the Alcohol Use and Drug Use Scales [63], a Habits Inventory, and the SCID. Data on services used and other societal costs (e.g. criminal/legal, public assistance) will be obtained with the Services Utilization and Resources Form for Schizophrenia (SURF) [64]. The SURF is a multi-item form that uses participants’ or caregivers’ report to document comprehensively the number, type, and duration of health services and consumption of non-health resources, such as criminal justice events and public assistance. This form has been adapted for use with the younger population in this study. The Premorbid Adjustment Scale (PAS) [65] will be acquired at baseline to assess preexisting differences in functioning. A brief selection of neuro- and social cognitive measures from the MATRICS battery will be used.

Evaluating the impact of ED within a year limits the significant attrition bias that tends to accrue over longer follow-up periods with this challenging population [66]. Indeed, one of the outcomes that are hopefully impacted by ED will be the quality and duration of engagement with care. The Service Engagement Scale [67] will help assess the domains of availability, collaboration, nature of help seeking and medication adherence.

Table 2 depicts assessment timeline. Further clinical assessment (at STEP/PREP^R^) will include routine physical and neurological examination with an initial work-up for common ‘secondary’ causes of psychosis that will include blood tests for a complete blood count, standard electrolyte and hepatic panels, thyroid function and urine toxicology and, when clinically indicated, testing for syphilis and HIV and structural brain imaging.

**Table 2:** **Research evaluation procedures and timeline**

**Months after enrollment in FES**	**0**	**6**	**12**
SIPS	x		
SOS & Prescription Medication Log	x*		
Pathways to Care Interview	x		
SCID I, selected SCID II sections	x		x
Demographics, Medical History	x		
Neurocognitive & Social cognitive battery:	x	x	x
1. premorbid IQ estimate- WRAT-4 reading (*only at baseline)*			
2. processing speed: MATRICS symbol digit			
3. verbal learning: MATRICS Hopkins verbal learning test			
4. social cognition: MATRICS- MSCEIT			
Habits inventory, AUS/DUS, Cannabis Scale	x	x	x
PANSS, Calgary Depression	x	x	x
Premorbid Adjustment Scale	x		
Global Functioning: Social and Role	x	x	x
Service Utilization and Resources Form (modified)	x	x	x
Service Engagement Scale (SES)		x	x
LUNSERS medication side effect scale	x	x	x
GAF, Heinrichs QOL, SF-36 Health Survey	x	x	x
Suicidality (C-SSRS), Aggression (MOAS)	x	x	x

*repeated within one month if confidence in DUP estimate is “possible” or “probable”.

Raters at each site will be trained using a set of common vignettes developed from historical cases at each site. Reliability assessment will focus on accurate measurement of DUP and pathways to care. Regular consensus calls will determine the team’s level of confidence (definite, probable or possible) in psychosis onset date from the SOS and offset date from the Prescription Medication Log. These measures will be repeated at 1 month when confidence in the initial estimate of DUP is only “possible” or “probable”. Ongoing monitoring of inter-rater reliability, as often as every three months for the key measures, will help reduce drift among raters and between sites.

### Statistical considerations

#### Choice of primary and secondary outcomes

The primary outcome of this study is DUP2. This is related to our primary aim of shortening time to entry into an FES with established effectiveness. We consider DUP1 a more direct measure of the ‘demand’ contributors to overall DUP, while the difference (DUP2-DUP1) related more directly to ‘supply’ side contributors.

The secondary aim of this study is to determine if DUP reduction results in improvements in clinical state at enrollment and improvements in treatment engagement and functional outcomes above and beyond that afforded by an FES. There is no evidence of a threshold at which DUP becomes significantly more determinative of outcome [68]. Analyses from different cohorts have suggested that efforts be made to shorten DUP to <1 month [69] or <3 months [70] but these differences likely result from heterogeneity in the samples and nature of the outreach efforts. Given this, we will not use an arbitrary threshold to adjust for baseline DUP (that could post-hoc make our FES’ look better or worse), but instead used linear regression across the full range of baseline DUPs to interrogate mediation effects on differential outcomes across the 2 sites.

#### Sample size and power

Since there is no conventional closed-form to estimate the statistical power for this type of design, we applied a flexible method to simulate the power empirically [71]. 1000 simulation datasets were generated by drawing random examples based on the alternative hypothesis of the design. The proposed analysis was then performed in each simulated dataset. The empirical power is the fraction of p values which are smaller than 0.05.

Extant measures of DUP are known to have a non-normal distribution, and the natural log-transformation will be used to estimate sample size. Our primary hypothesis is that the ED campaign will effectively decrease DUP at STEP vs. PREP (non-ED site). Based on historical data from 2006–2013, the estimated geometric means and standard deviations were 3.7 ± 3.7 months (corresponding to 1.3 ± 1.3 in a natural log scale) for PREP and 5.0 ± 3.7 months (corresponding to 1.6 ± 1.3 in natural-log scale) for STEP. We project that DUP will remain within the same range at PREP during years 2 through 4, but will decrease from 1.6 to 0.8 in the natural log scale at STEP (ED site). This effect size corresponds to a 55% decrease in the geometric mean DUP (months), and is comparable to the TIPS I study (1997–2000, n = 281) that reduced DUP to a median of 5 weeks [range, 0–1196 weeks] from 16 weeks [range, 0–966 weeks]. To power our study, we include historical DUP data from PREP (n = 44) and STEP (n-99) into the pre-campaign group. We plan to enroll 22 more subjects at PREP and 34 more at STEP during the first year (pre-campaign, as the baseline control for each site), as well as 66 participating at PREP and 102 participants at STEP during years 2, 3, and 4 (post-campaign). With the sample sizes of 66 (44 plus 22) at PREP and 133 (99 plus 34) subjects at STEP during the pre-campaign period, and 66 at PREP and 102 at STEP during the post campaign period, we will have 81% empirical power to detect a difference of 0.8 in the change of log-DUP from pre to post period between two sites at 0.05 alpha level.

#### Analysis plan

Patient characteristics will be compared between sites and between periods. The primary analysis will be two-way analysis of variance (two-way ANOVA) that includes two categorical explanatory variables both with two levels: period (“pre campaign” & “post campaign”) and site (“STEP” & “PREP^R^”). The combination of historical data since 2006 and the first year outcome data for each site will serve as the reference to estimate change in DUP (in natural log scale) at each site. The primary hypothesis is the ED campaign will result in greater decline in DUP at STEP compared to PREP^R^. The interaction of period and site will be used to test this hypothesis. This test is of primary interest for our study, although linear contrasts will be also conducted to compare between-site differences for the pre- and post-campaign periods. A significant interaction effect between period and site will disconfirm the null hypothesis and show that the outcome varies between pre and post campaign periods depending upon the employment of early detection. We do not anticipate significant main effects, but a significant study period effect would indicate a pre vs. post study difference on average across sites and a significant site effect would indicate that one site had a greater DUP on average across study periods. In addition, adjusted analyses will be performed with inclusion of covariates. Potential confounders such as participants’ age, gender, race/ethnicity will be included in the adjusted analyses.

A supportive analysis will investigate any effect of time by including the fixed effect of year. Other secondary analyses will include modeling of the observed percentiles of DUP (e.g., quartiles in months) using quantile regression, with the same main and interaction effects as in the approach of primary analysis. Overall, we chose our primary analysis to reflect an investigation of perhaps the most clinically meaningful change in DUP (decrease from pre period at STEP), whereas our supportive analysis will give us more insight into how early detection affects DUP across the range of DUPs observed during the pre period.

Other analyses related to our secondary aim, will interrogate whether and to what extent DUP reductions predicted improvements in clinical presentation, treatment engagement and measures of symptomatic and functional recovery.

Statistical significance will be established in all analyses at alpha = 0.05 and analyses will be performed using SAS 9.3 (Cary, NC).

## Discussion

This study will adapt and test an approach to early detection that will employ several innovations in design, delivery and measurement highlighted below:

1. Innovating with the end in mind. The project leverages existing public-academic collaborations to implement a catchment-based approach to early detection. This will allow evaluation of scalability across similar sectors of the state and country for a given value (outcomes/cost) [72]. STEP and PREP^R^ are each integrated, within 2 of 50 nationwide State Mental Health Agencies that constitute a de facto national mental health system, reaching into nearly every community in the country and serving patients in the public sector, with serious mental illnesses. While states differ considerably in the level and mechanisms of funding, the presence of a 50 + year old infrastructure in the public sector with experience in the care of severe and persistent mental illnesses provides a natural framework within which to build a national approach [18].
2. The use of social marketing has not to our knowledge been previously attempted for improving access to mental health care. This involves the adaptation of commercial marketing approaches towards behavior change that will be applied in the service of public health goals. A variety of stakeholders (parents, friends, professionals and young patients) whose behavior this campaign will seek to influence, will be engaged to develop and test messages. Online analytic tools will be used to track the uptake of messages and refine the campaign and represents an advance over traditional, static mass media messaging. Furthermore, the leveraging of social media to target adolescents and young adults will we hope deliver more effective and age appropriate ways to engage a population well known to resist entry into mental healthcare systems which have been traditionally oriented towards older, chronically ill patients. The details of the resulting campaign will be described in a forthcoming paper.
3. The project takes a deliberately ‘interventionist’ stance to assessing local pathways to care i.e. the mounting of a campaign targeting presumed sources of DUP is expected to reveal more actionable information about these sources of delay and other salient features of local pathways. This is in contrast to surveying the community for barriers that may not reliably predict those that are actually operative in determining DUP. This is in keeping with our model of DUP as resulting from a more dynamic interplay between demand and supply contributors.

Pathways to care for each patient will be systematically assessed using structured interviews of patients, caregivers and clinicians and reviews of clinical documents. Barriers of greatest salience (magnitude and/or malleability) are presumed to vary across the heterogeneous U.S. landscape. Our use of a measure that will allow generation of locally detailed maps will allow us to describe and respond to the sometimes exquisitely local barriers that characterize U.S. pathways to care. While TIPS focused on enhancing mental health literacy on the one hand and making FES more rapidly responsive to referrals on the other, it took for granted the existing institutional structures that defined the pathways within which patients and caregivers sought care. By taking a more agnostic stance on pathways and assessing in detail events and actors in both the ‘demand’ and ‘supply’ sides of DUP we will be able to provide information on how these individuals and organizations perform as a network of care and suggest sustainable ways to change them and sustain DUP reductions beyond the life of the campaign.

In summary, testing an empirically based DUP reduction strategy (Primary Aim) is a logical next step for both FES programs (STEP & PREP^R^) as we seek effective ways to hasten access to our established and effective services. Also, demonstrating the added value of successful early detection (ED) to an existing FES (STEP) in a controlled manner (Secondary Aim) will inform models of how best to resource and implement a comprehensive approach to secondary prevention for psychotic disorders in the U.S. Given this project’s integration within a public-academic collaboration, the results will be primed for national dissemination.

## Abbreviations

TIPS: Early treatment and intervention in first episode psychosis
DUP: Duration of Untreated Psychosis
FES: First-Episode Service
EI: Early Intervention
ED: Early Detection
STEP: clinic for Specialized Treatment Early in Psychosis
PREP^R^: Prevention and Recovery in Early Psychosis
CMHC: Connecticut Mental Health Center
MMHC: Massachusetts Mental Health Center
PEC: Public Education Campaign
POD: Professional Outreach and Detailing
RAS: Rapid Access to STEP
